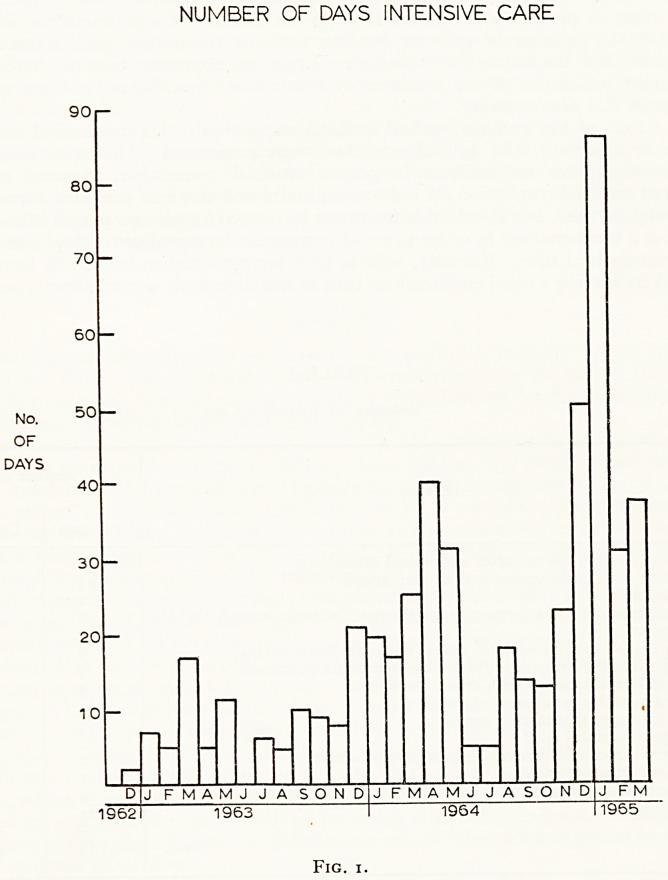# Is an Intensive Care Unit Really Necessary?

**Published:** 1965-10

**Authors:** Peter J. F. Baskett

**Affiliations:** Senior Registrar in Anaesthetics, United Bristol Hospitals and Frenchay Hospital Bristol


### IS AN INTENSIVE CARE UNIT REALLY NECESSARY?

BY

PETER J. F. BASKETT, B.A., M.B., B.CH., B.A.O., F.F.A.R.C.S.

*Senior Registrar in Anaesthetics, United Bristol Hospitals and Frenchay Hospital Bristol.*

Recent advances in respiratory and cardiovascular resuscitation have enabled a number of patients to be saved who in the past would have succumbed. However, these advances have only been made possible in practice by dint of intensive medical care and nursing by highly trained persons using complicated, delicate, and expensive apparatus. The application of this sort of care in a general ward presents a great many drawbacks. As a result of intensive care being devoted to one patient in the ward, the rest of the patients may get but scant nursing attention and are generally disturbed by the noise of machines and the frequent visits of doctors. A great deal of space is required around the bedside to allow for bulky respirators, suction apparatus, oxygen cylinders, E.C.G. oscilloscopes, etc., and usually at least two bed spaces are occupied. Since piped oxygen and suction are not universally available in general wards, nursing the patient is particularly difficult, with cylinders and suction machines getting in the way. Delicate and expensive apparatus often gets damaged and vital pieces get lost by transfer from ward to ward, and from use by inxeperienced people. It is impractical and uneconomical to have enough of this specialized equipment for every ward, and one doctor finds it difficult to supervise the intensive care of several patients scattered throughout the hospital. There may not be sufficient nursing staff on duty in any particular ward who are experienced in work of this nature, and in addition cross infection is a great problem, particularly in patients with tracheostomies.

It is for these reasons that there has been a gradual realization during the past decade of the need for an Intensive Care Unit in every major general hospital (Fairley, 1961; Editorial, 1961; Gardner, 1964). Ideally, this is a unit containing a small number of beds where every patient both needs and receives intensive care. The unit is equipped with all apparatus for respiratory and cardiovascular monitoring and resuscitation, and is staffed by highly trained nurses who are experienced in this work and who understand and are able to manage the apparatus. This type of unit necessitates a large number of nurses for a relatively small number of patients, but even so is more economical with nursing staff than if the patients are scattered throughout the hospital.

In order to assess the requirements of a unit in the United Bristol Hospitals, the members of the Anaesthetic Department decided in December 1962 that they would compile a brief record of all patients who received intensive care in the general wards from members of the Department, and who would have benefited by treatment in an Intensive Care Unit.

In each case the nature of the illness, the type of treatment provided, the minimum duration of care required, and the outcome, were recorded. Patients who were recovering from open heart surgery are not included in this report as they were treated in the Cardiac Unit which provides intensive care for these cases.

### THE PATIENTS TREATED

During the period December 1962–March 1965, members of the Anaesthetic Department assisted in the intensive care of 143 patients. Of these, 127 were in the Bristol Royal Infirmary, 10 in the Bristol General Hospital and 6 in the Bristol Royal

Hospital for Sick Children. These figures indicate that the siting of an Intensive Care Unit should be at the Bristol Royal Infirmary.

It is essential that a patient who has been receiving treatment in an Intensive Care Unit should return to his own ward as soon as he is deemed fit to do so. This vacates a bed in the unit for someone in need, and at the same time prevents the patient who is recovering, from being surrounded by those who are very seriously ill. Since the mortality rate in this type of unit in inevitably high, it is obviously not a good place for the morale of patients who are well enough to take an interest in the progress of others.

The duration of treatment for these 143 patients averaged just under 3·6 days, with a range of six hours to five weeks. Altogether a total of 521 days of intensive treatment was provided.

NUMBER OF DAYS INTENSIVE CARE

Fig. 1.

Figure 1 shows the number of days of intensive care provided monthly over the period of study.

While there was considerable variation in the amount of treatment required from month to month, it is apparent that there has been a definite increase during the the period of study. In the first 12 months, December 1962–November 1963, 44 patients were treated, occupying a period of 85 days of intensive care; in the last 12 months, April 1964–March 1965, 63 patients were treated, occupying a period of 352 days. It is difficult to account for the longer average period of intensive care required for patients during the last 12 months. Part of the difference lies in the fact that more patients with chronic bronchitis associated with respiratory failure are now being treated, and these cases, being balanced on a knife-edge, tend to take a long time to get completely weaned off a respirator. Two cases of myasthenia gravis requiring artificial respiration for several weeks fell into the second period.

In order to provide some guidance as to the amount of equipment required, we recorded the number of patients needing artificial respiration with a mechanical respirator, and the number of patients requiring tracheostomy, because these factors give some indication of the numbers of respirators, humidifiers, and gas analysis apparatus that are necessary.

Of a total of 143 patients, 90 had artificial respiration with a mechanical respirator (Cape or Cyclator), and 49 had a tracheostomy performed. The usual reason for tracheostomy was to facilitate long-term artificial respiration. Several patients required artificial respiration for only 12–24 hours and this was provided through an endotracheal tube, but should this treatment be needed for a longer period it is wise to perform a tracheostomy in order to avoid permanent laryngeal or tracheal damage by the endotracheal tube. Recently, infants with laryngo-tracheo-bronchitis have been treated by leaving a nasal endotracheal tube *in situ* to provide a good airway, access to

TABLE I

Reasons for Intensive Care

| Disease | Number of | |
|---|---|---|
| | Patients | Deaths |
| Cardiorespiratory failure after abdominal operations | 36 | 14 |
| Poisoning with barbiturates and similar drugs | 25 | 4 |
| Head injuries | 14 | 8 |
| Acute respiratory failure superimposed upon "chronic bronchitis" | 8 | 4 |
| Chest injuries | 7 | 1 |
| Post-operative cardiac surgical cases (not open heart surgery) | 6 | 4 |
| Laryngo-tracheo-bronchitis ± bronchopneumonia in infants | 5 | 0 |
| Pharyngo-laryngectomy with colon transplant | 5 | 2 |
| Cerebro-vascular accident with apnoea | 4 | 4 |
| Exacerbation of myasthenia gravis | 3 | 0 |
| Damage to cervical cord | 3 | 3 |
| Coronary thrombosis progressing to cardiac arrest | 2 | 1 |
| Prolonged status epilepticus | 1 | 0 |
| Spontaneous hypothermia (elderly patients in cold winter) | 2 | 0 |
| Miscellaneous | 22 | 3 |
| Totals | 143 | 48 |

secretions, and a route for artificial respiration (McDonald and Stocks, 1965), techniques which have been developed in Australia and Liverpool. So far, in a limited number of cases, we have had good results with this, and the method seems to represent a considerable advance because the management of a tracheostomy in a neonate or infant is notoriously difficult.

The illnesses for which intensive care was given are shown in Table I. The largest group consisted of patients who developed some form of circulatory or respiratory failure or both after an abdominal operation, such as residual respiratory paralysis following the administration of a relaxant drug, or acute cardiac failure. Many of these patients require only a short period of intensive care, and could be adequately dealt with in a recovery ward adjoining the theatre suite, if it were available.

Overdosage with barbiturates to the point of causing respiratory or cardiovascular depression formed the next largest group. These tended usually to be short-term problems as far as intensive care was concerned, but depending on the amount and type of barbiturate ingested this care may be required for up to 10 days. If a tranquillizing drug such as phenothiazine or a monoamine-oxidase inhibitor had also been taken, cardiovascular disturbances tended to be more pronounced. This group included several children who took these drugs in mistake for sweets.

It is perhaps surprising that more cases of myocardial infarction have not been treated, but it is likely that so far these cases have been managed by physicians alone, without anaesthetists being involved. If an Intensive Care Unit were available, it should be ideal for the management of these patients, who are in imminent danger of progressing to ventricular fibrillation.

### MORTALITY

Of the 143 patients who received intensive care 48 died, a mortality rate of approximately one third. This is in agreement with experience in other centres (Gardner, 1964; Pearce, 1961). In addition, two patients, both with severe head injuries, are living but with gross cerebral damage due to the injury.

Good results have been obtained in cases of barbiturate or similar drug poisoning, myasthenia gravis with respiratory failure, chest injuries, and laryngo-tracheo-bronchitis in infants. The results in head injuries are disappointing, but these are cases in which intracranial decompression was judged to be of no value—the symptoms and signs being due to brain laceration. No patient with a head injury or cerebro-vascular accident which progressed to apnoea survived. It is doubtful if putting such patients on a respirator performs any useful function.

It is not possible to say whether any of the patients who succumbed after intensive care in the general wards would have survived if they had been treated in a special unit. Nevertheless there have been several in which it was felt that the employment of nurses experienced in this work, combined with on-the-spot medical supervision and all necessary equipment, might have made the difference between failure and success. However, it is possible to say that morbidity could be reduced in an Intensive Care Unit (Edwards *et al.*, 1965). This applies particularly to the care of tracheostomies and the prevention of chest infections. Accidents with respirators should be reduced to a minimum in a unit in which the use of these machines is a routine matter. Cardiac arrest, which is ever likely in these patients, is more expertly managed in a small unit staffed by experienced persons with all necessary equipment to hand. Experience in the Cardiac Unit of the Bristol Royal Infirmary, which is a small unit giving specialized care, confirms these views.

### SIZE OF INTENSIVE CARE UNIT REQUIRED

It has been generally agreed that a unit of six beds in the Bristol Royal Infirmary is the size required. During the period of study, no more than five patients at any one time were being given intensive care, and six beds may seem more than adequate when judged entirely on the figures presented here. However, it would be foolish when planning the capacity of such a unit not to allow for expansion in this field. That expansion is taking place is demonstrated in the record of the increasing number of cases and patient days. In addition to the cases reported in this paper, patients have been given intensive care by members of other specialties, and anaesthetists have not been required. Since the anaesthetist has not, and has no wish to have, the sole management of all patients requiring intensive care, provision must be made for these additional cases when the size of the unit is planned. If an intensive care unit were available, there is no doubt that many patients with diseases that have not appeared in this report would be admitted to it—for example, patients suffering from bronchitis and bordering on respiratory failure requiring controlled oxygen therapy and regular blood gas analysis.

Ideally, an intensive care unit should be divided into areas in which "clean" and "dirty" cases are entirely separate. This, unfortunately, tends to be impracticable in a unit of only six beds, since it demands a large nursing staff, and presents other difficulties, not the least of which are architecture and finance. However, these difficulties can be largely overcome by allotting a special cubicle for "dirty" cases, and maintaining a rigid regime to minimize cross-infection. Such a system is infinitely better than attempting intensive care in a general ward.

### *Addendum*

An intensive care unit of six beds has now been planned for the Bristol Royal Infirmary, and is due to open in the autumn of 1965.

### *Acknowledgements*

I should like to express my thanks to Dr. G. L. Feneley and to Dr. John Clutton-Brock who gave invaluable help in the preparation of this paper; to the Registrars and S.H.Os. of the Department of Anaesthetics in the United Bristol Hospitals, who recorded details of the intensive care provided; to Mrs. Mary Heal who prepared the typescript and to Mr. F. E. Badrick who was responsible for the histogram.

### REFERENCES

Editorial (1961) *Anaesthesia*, **16**, 265.
Edwards, F. R., Richardson, J. C., and Ashworth, P. M. (1965) *Lancet*, **1**, 855.
Fairley, H. Barrie (1961) *Anaesthesia*, **16**, 267.
Gardner, E. (1964) *Anaesthesia*, **19**, 128.
McDonald, I. H. and Stocks, J. G. (1965) *Brit. J. Anaesth.*, **37**, 161.
Pearce, D. J. (1961) *Anaesthesia*, **16**, 308.

## Figures and Tables

**Fig. 1. f1:**